# Measuring Development of Self-Help Organizations for Patients with Chronic Health Conditions in Hong Kong: Development and Validation of the Self-Help Organization Development Scale (SHODS)

**DOI:** 10.3390/ijerph18031351

**Published:** 2021-02-02

**Authors:** Steven Sek-yum Ngai, Shan Jiang, Chau-kiu Cheung, Hon-yin Tang, Hiu-lam Ngai, Yuen-hang Ng

**Affiliations:** 1 Department of Social Work, The Chinese University of Hong Kong, Hong Kong, China; shanjiang@link.cuhk.edu.hk (S.J.); hytang@cuhk.edu.hk (H.-y.T.); ngaihiulam@link.cuhk.edu.hk (H.-l.N.); yhng@swk.cuhk.edu.hk (Y.-h.N.); 2 Department of Social and Behavioral Sciences, City University of Hong Kong, Hong Kong, China; ssjacky@cityu.edu.hk

**Keywords:** self-help organization, assessment of organizational development, psychometric properties, confirmatory factor analysis, measurement tool

## Abstract

Self-help organizations (SHOs) enable patients with chronic health conditions (PCHCs) to overcome common difficulties through the exchange of knowledge and mutual assistance, which serves as the basis for promoting the self-reliance and well-being of PCHCs. Nevertheless, practical challenges persist because little is known about what and how to evaluate for the developmental outcomes of SHOs. To address this knowledge gap, the present study seeks to develop and validate the Self-Help Organization Development Scale (SHODS). A total of 232 core members from 54 SHOs in Hong Kong participated in our study. The SHODS structure was validated by confirmatory factor analysis. This analysis derived five factors: citizen support, business support, member recovery and mutual aid, organizational health, and functional sustainability. The five-factor structure demonstrated stability across various types of SHOs, as validated by the subgroup analysis based on two criteria: duration of SHO establishment and organization affiliation. Good concurrent validity was supported by significant correlations between the SHODS factors and organizational variables, including staff supervision, staff understanding, networking, advocating, and educating the public and patients. The SHODS also showed excellent internal consistency. In conclusion, the SHODS is a psychometrically sound instrument for measuring the developmental outcomes of SHOs.

## 1. Introduction

### 1.1. Background: Significance and Challenges of Self-Help Organizations

Chronic health conditions such as arthritis, diabetes, and heart disease are commonly defined as recurrent health-related states lasting above one year [1]. Globally, the prevalence of chronic health conditions is growing, undermining the well-being of individuals and presenting a great burden to society as a whole [2,3]. The Institute for Health Metrics and Evaluation indicated that the global burden of chronic health conditions rose by 40 percent from 1990 to 2017 [4]. The social costs of chronic health conditions, such as the loss of human resources, also have a negative impact on the economic development of a society [5]. Thus, it is essential to reduce the incidence of chronic diseases and improve the quality of life of patients with chronic health conditions (PCHCs). 

Self-help organizations (SHOs) are formed when people with a common problem (e.g., PCHCs) come together to share their perspectives and knowledge in problem-solving, often with support from professionals such as social workers and medical professionals [6,7]. The concept of self-help creates opportunities for service users—including PCHCs and their family members—to gain a better understanding of chronic health conditions and obtain unique forms of help from mutual aid processes [8]. Members can express their feelings, communicate with and support each other, and find ways to solve problems together in a safe environment free from discrimination because members have common experiences or face similar difficulties [6]. By establishing relationships, sharing leadership, engaging in mutual assistance, and gaining knowledge, participants of SHOs can achieve empowerment and regain self-efficacy, which improves their confidence to address their current challenges [9]. These organizational practices are particularly important for disadvantaged groups—such as people with chronic health conditions—who are less likely to express their needs and demands [9]. 

Despite the importance of SHOs to PCHCs, SHOs face challenges in their survival and development. Financial stability is important for sustaining an SHO, but it may face financial challenges [10]. SHOs need to find ways to access and attract resources necessary to their viability as well as adapt to ongoing changes in their environment [11]. Moreover, it may be challenging to gain public support, and stigmatization often arises due to the community’s lack of knowledge about the chronic health condition [12]. Member non-participation and withdrawal is another challenge for SHOs. Members may withdraw from the SHO if they feel that it is no longer necessary to participate in the organization, for instance, when the SHO does not meet their needs and expectations, or when issues of group dynamics, like disagreement among members, arise [10,13]. Additionally, there is a lack of measurement tools to systematically and comprehensively assess the multiple aspects of SHO development. Thus, there is an imperative need to develop frameworks and instruments that can be utilized to help SHO leaders, managers, and related professionals evaluate and promote SHO development.

### 1.2. The Present Study

Hong Kong is a city where the East meets the West. The majority of Hong Kong’s population is Chinese [14], and its Chinese culture is combined with its history as a British colony. In Hong Kong, the first SHO for patients with disabilities was established in 1964, and the first SHO for patients with chronic illness was established in 1979 [15]. Since then, the number of SHOs for PCHCs in Hong Kong has increased rapidly. It has been estimated that over 170 SHOs address chronic health conditions in Hong Kong today [16]. Despite the rapid proliferation of SHOs in Hong Kong, their development has stabilized but also stagnated, and they face many challenges in their further development. These challenges include difficulties in leadership succession, a shift in focus from mutual support to being more welfare-focused, issues in recruiting new members, weak group identification, and lack of public awareness about the self-help approach [17]. The practical challenges of SHO development persist because little is known about what and how to evaluate the developmental outcomes of SHOs.

Given the considerations mentioned above, including the importance of SHOs, challenges to SHO survival, and the scarcity of empirically validated assessment tools for SHO development, this study aims to: (1) propose an integrated framework that provides a comprehensive perspective for conceptualizing and assessing SHO development; and (2) develop and validate the Self-Help Organization Development Scale (SHODS), which would measure the developmental outcomes of SHOs.

### 1.3. Model Development: Indicators of SHO Development

Currently, there is a lack of a comprehensive framework for understanding the development of SHOs, so the first task is to identify indicators to evaluate the developmental outcomes of SHOs. In view of the similar features of SHOs and nongovernmental organizations (NGOs), the theory related to NGO development may be useful in the absence of theories directly related to SHOs. 

The following theoretical models have been widely used to evaluate organizational development: the goal attainment model, the internal process model, and the system resource model [18,19]. The goal attainment model is a prevailing model that focuses on whether an organization’s specific goals and objectives are being achieved [18]. The internal process model focuses on the communication processes among internal stakeholders that are essential to the organization maintaining itself as a social unit [18]. In this perspective, an organization’s intrinsic characteristics, like organizational health and stability, can be used as indicators to measure organizational development. The system resource model emphasizes the interdependence between the organization and its external social system; it focuses on how an organization acquires support and various resources from its social environments [18]. 

In general, most existing research has focused on the outputs of SHOs—that is, the influence of SHOs on their members’ rehabilitation—but validated indicators related to organizations’ internal and external development are scarce. It should be noted that relying solely on the goal attainment model is not sufficient for comprehensively capturing the developmental outcomes of SHOs. In response to these limitations, we integrated the three models mentioned above to provide a comprehensive framework that can be used to assess SHO development.

Based on the literature review on local and nonlocal studies about SHO development, we propose a five-factor framework that includes five aspects of SHO development: member recovery and mutual aid, functional sustainability, organizational health, citizen support, and business support [8,18,20,21,22,23,24,25,26,27,28]. 

The common goal of SHOs is to help members develop forms of mutual assistance and recover from the chronic health condition as much as possible. In the goal attainment model, member recovery and mutual aid and functional sustainability were proposed for evaluating whether SHOs achieve their goals of mutual aid and effectively meeting members’ needs. The factor of member recovery and mutual aid specifically refers to the positive change in members’ sense of hope and resilience in the face of the chronic health condition, the mutual aid shared among members, reduced stress, and increased encouragement among members [23,24]. Functional sustainability refers to the SHO’s achievements in increasing members’ knowledge about the chronic health condition and their self-care, satisfying members’ needs, and having members affirm their own values [8,21,24,27]. 

In addition, guided by the internal process model, we developed another factor, organizational health, to evaluate an organization’s inherent ability to maintain its stability, seen in SHOs’ financial stability, stability of resources, and adaptability to the changing environment [8,21,24,26]. 

Furthermore, following the system resource model, we proposed two final factors—citizen support and business support—to assess the organization’s ability to obtain support from different stakeholders. Specifically, citizen support refers to citizens’ understanding of, monetary support for, and supportive actions (such as volunteering and advocating) for the SHO [20,24,25,28]. Furthermore, SHOs often liaise with businesses; hence, it is essential to have good working relationships with businesses in order to share expertise and networks [20,21,22,24,28]. Thus, support from business resources can be used to evaluate SHOs’ development. Business support refers to businesses’ understanding of the SHO, good relationships between the SHO and businesses, and whether businesses support the SHO (e.g., providing financial support). 

Altogether, this framework includes both external factors involving interactions with the macro social system (i.e., citizen support and business support) and internal factors related to the SHO’s innate ability to maintain its stability (i.e., organizational health). It also includes the outcomes and outputs of SHOs, evaluated by member recovery and mutual aid, as well as functional sustainability. These five factors are expected to be significantly and positively related to one another. The five factors constitute our theoretical model of SHO development. With such a framework, this study aims to create a measurement tool for assessing SHOs’ development. 

### 1.4. Concurrent Validity: Organizational Variables and SHO Development

Due to the lack of measurement tools for SHO development, little is known about the concurrent validity of those measurement tools in this respect. Therefore, organizational variables were considered to be correlated with SHO development for the test of concurrent validity. Effective organizational practices can enhance SHO development [18,19]; such practices include managing staff and interacting with external stakeholders in the community [18,19]. Hence, we identified key components from the existing literature for assessing these organizational practices and derived five major organizational variables. 

Staff supervision and staff understanding are key organizational variables of staff management [18,29]. Staff supervision refers to committee members’ guidance to staff and evaluation of staff performance, including regular supervision, executive committee members giving clear instructions to staff, regular staff evaluation, provision of opportunities for staff members to exchange ideas, and provision of a safe working environment for staff [29]. Staff understanding refers to staff understanding their own roles and responsibilities in the SHO, and staff understanding the self-help and mutual aid vision of the SHO [29]. These staff management practices can enhance staff collaboration and participation, support the staff, and boost organizational morale through staff empowerment [18]. Thus, effective staff management is assumed to be positively associated with SHOs' developmental outcomes.

Networking, advocating, and educating the public and patients are key organizational variables that indicate effective interaction with external stakeholders [18,24,28,30]. Networking refers to the engagement of community resources and external stakeholders, which includes the establishment of networks or coalitions with stakeholders such as other nonprofit organizations, other SHOs, funders, media, businesses or volunteer groups, university students, and professional advisors. Networking also includes reaching out to community resources such as applying for government funding, promoting various community stakeholders to become community partners, and connecting with patient resource centers to receive resources to facilitate the operation of the SHO [24,25,28]. *Advocating* refers to policy, welfare, and community changes the SHOs have advocated. Policy changes include liaising with the government to improve medical and rehabilitation policies. Community changes include advocating for community facilities and supportive services [28,30]. Educating the public and patients refers to promoting mutual aid spirit to the public and patients. It also refers to increasing the public’s knowledge about SHOs, SHO’s services and needs, and chronic health conditions, and reducing stereotyping and stigmatization toward people with chronic health conditions [24,28]. These practices can potentially create new opportunities for the SHOs by developing mutual understanding and fruitful collaboration with external stakeholders, refining SHO operation in response to changes in the external environment, and introducing innovative insights that enhance SHO development [18]. 

Collectively, these organizational variables are hypothesized to be significantly and positively related to the five SHO development factors. Thus, they are used as criteria to evaluate the concurrent validity of the SHODS developed in this study. Based on the above review, we propose the following hypotheses:

**Hypotheses 1** **(H1.)** *Staff supervision is positively correlated with SHO development factors.*

**Hypotheses 2** **(H2.)** *Staff understanding is positively correlated with SHO development factors.*

**Hypotheses 3** **(H3.)** *Networking is positively correlated with SHO development factors.*

**Hypotheses 4** **(H4.)** *Advocating is positively correlated with SHO development factors.*

**Hypotheses 5** **(H5.)** *Educating the public and patients is positively correlated with SHO development factors.*

## 2. Materials and Methods

### 2.1. Procedure and Participants

With assistance from a local NGO that supports and coordinates SHOs, we collected a total of 232 complete questionnaires from core members of 54 SHOs in Hong Kong who play important roles in SHO operation and development; this is approximately 31.8 percent of the total number of SHOs for PCHCs in Hong Kong today [16]. Informed consent was obtained from all participants. These participants were primarily executive committee members (52.6%) and chairpersons and vice-chairpersons (27.1%). The participants’ average tenure in office was 81.4 months. Most participants had education levels of secondary school or above (84.3%). The largest age group was 60–69 years old (33.5%), followed by 50–59 years old (24.8%). There was an approximately equal proportion of male (47.0%) and female (53.0%) participants. 

### 2.2. Measures

#### 2.2.1. Development of Self-Help Organization Development Scale (SHODS)

The measure used in our survey was developed by adapting a variety of existing scales and theories from the literature [8,18,19,20,21,22,23,24,25,26,27,28]. Accordingly, items drawn from the major indicators of SHO development were initially identified in the literature and incorporated into a questionnaire as a measure for the study. 

As a check for face validity and clarity of the items, five core SHO members participated in a pilot study, and their feedback was incorporated to refine the items in the scale. Subsequently, the scale was further modified and confirmed by two university professors and four social workers from an NGO that supported us in data collection. 

The final SHO development scale consisted of 18 items, divided into the categories of citizen support, business support, member recovery and mutual aid, organizational health, and functional sustainability. The items asked about the SHO in relation to these factors in the previous six months. The items adopted a five-point scale, with 1–none, 2–relatively less, 3–moderate, 4–relatively more, and 5–a lot. The scale was converted into a range from 0 to 100, with 0 representing the lowest level (none) and 100 representing the highest level (a lot), for easy interpretation and comparison. Reliability was assessed by computing Cronbach’s alpha for each item.

#### 2.2.2. Measurement of SHO Development Factors

Citizen support (CS) consisted of four items measuring citizens’ support for the SHO. Sample items include “Receiving donations from citizens” and “Citizens collaborating with the SHO to advocate for patient rights.” Higher scores indicate that the SHO receives more citizen support.

Business support (BS) was measured with three items related to the relationship with, and resources provided by, businesses/enterprises. Sample items include “Having good relationships with businesses and enterprises” and “Businesses and enterprises having a good understanding of the SHO.” Higher scores indicate that the SHO has more support from businesses and enterprises.

Member recovery and mutual aid (MRMA) was measured with four items asking about SHO members’ sense of hope and resilience in the face of their chronic health condition, mutual aid among members, alleviation of members’ stress, and increased encouragement among members. Sample items include “Good self-help and mutual support among members” and “Positive change in members’ sense of hope and resilience in the face of the chronic health condition.” Higher scores indicate better member recovery and mutual aid among members of the SHO.

Organizational health (OH) was measured with four items on the SHO’s financial stability, availability of various resources (e.g., human and material resources), and adaptability. Sample items include “Stable funding” and “Stable supplies, such as wheelchairs, computers, and recreational facilities.” Higher scores indicate that the SHO has better organizational health.

Functional sustainability (FS) was assessed with three items on the SHO’s achievement in terms of members’ increased knowledge of self-care and their chronic health condition, satisfying members’ needs, and members affirming their value in the past six months. The items are: “Members gain more knowledge about self-care and their chronic health condition,” “Members express fulfillment of their needs,” and “Members recognize their own values.” Higher scores indicate that the SHO has better functional sustainability.

#### 2.2.3. Measurement of Organizational Variables

To test for concurrent validity, the measurement tools of organizational variables were derived from existing reliable measures in published literature. The items asked about the SHO in relation to these variables in the previous year. The items for staff supervision, staff understanding, networking, advocating, and educating the public and patients used a five-point scale, with 1–none, 2–relatively less, 3–moderate, 4–relatively more, and 5–a lot. These scales were converted into a range from 0 to 100, with 0 representing the lowest level (none) and 100 representing the highest level (a lot), for easy interpretation and comparison. 

Staff supervision was measured with six items. Sample items include “Regular supervision of staff performance” and “Opportunities for exchange among staff members” [29]. This scale yielded a satisfactory Cronbach’s alpha of 0.873. Higher scores indicate better staff supervision.

Staff understanding was assessed with two items: “Staff understand their own roles and responsibilities in the SHO” and “Staff understand self-help and mutual aid vision of the SHO” [29]. The composite score of the two items yielded a satisfactory reliability Cronbach’s alpha of 0.805. Higher scores indicate greater staff understanding.

Networking was measured with ten items. Sample items include “Inviting health care professional consultant teams to provide professional advice to PCHCs and the SHO” and “Engaging different external stakeholders to become the SHO’s collaborative partners” [28]. The composite score of the ten items yielded a satisfactory Cronbach’s alpha of 0.854. Higher scores indicate more networking with external stakeholders.

Advocating was measured with five items. Sample items include “Promoting change in the government’s medical policies” and “Striving for community facilities for PCHCs” [28]. The composite score of the five items yielded a satisfactory Cronbach’s alpha of 0.901. Higher scores indicate greater engagement in advocacy.

Educating the public and patients was assessed with six items. Sample items include “Promoting a spirit of self-help and mutual aid in the community” and “Enhancing the community’s understanding of chronic health conditions” [28]. The composite score of the six items yielded a satisfactory Cronbach’s alpha of 0.904. Higher scores indicate more activity in educating the public and patients about SHOs or chronic health conditions.

### 2.3. Data Analysis

A confirmatory factor analysis (CFA) was conducted to validate the scale. We also conducted individual CFAs for two pairs of subgroups: SHO duration of less than or equal to 240 months vs. SHO duration of more than 240 months, using the median as a cutoff [31]; and whether the SHO is affiliated with another SHO/NGO vs. not affiliated. The reliability of the scale was examined with Cronbach’s alpha, and the concurrent validity of the scale was validated by correlating the SHODS factors with the organizational variables. 

Multiple fit indices were used to evaluate the goodness-of-fit of the model: Comparative Fit Index (CFI), Tucker-Lewis Index (TLI), Root Mean Square Error of Approximation (RMSEA), and Standardized Root Mean Square Residual (SRMR). We used the following criteria to identify whether a reasonable fit was reached: CFI and TLI above 0.90, RMSEA and SRMR below 0.08 [32]. All analyses were performed in SPSS 25.0 (IBM Corp., Armonk, NY, USA) and Mplus 8.1 (Muthén & Muthén, Los Angeles, CA, USA).

## 3. Results

### 3.1. Scale Items and Descriptive Statistics

The SHODS consisted of five factors: Citizen Support (CS), Business Support (BS), Member Recovery and Mutual Aid (MRMA), Organizational Health (OH), and Functional Sustainability (FS). The descriptive statistics of the five factors are shown in Table 1.

### 3.2. Confirmatory Factor Analysis

**Figure 1:**
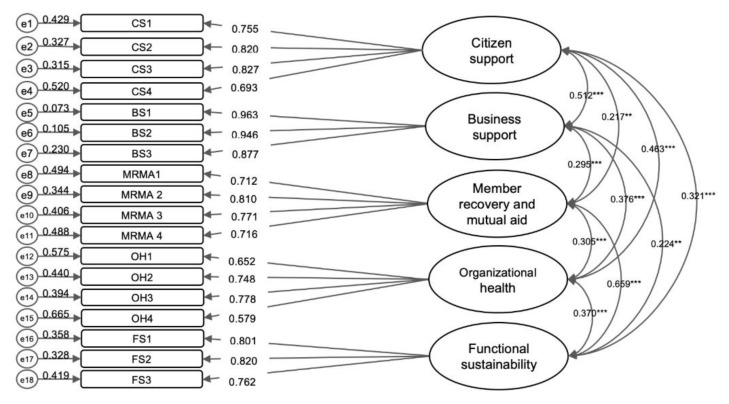
Factor Structure of the SHODS, ** *p* < 0.01, *** *p* < 0.001.

The results of the CFA indicated a good fit for the five-factor structure of the SHODS: CFI = 0.959, TLI = 0.950, RMSEA = 0.056, SRMR = 0.052. The standardized factor loadings of each factor are presented in Figure 1. As shown, all the factor loadings were above 0.5. 

We further conducted subgroup analysis based on two criteria: duration of SHO establishment in number of months (using the median as the cutoff), and organization affiliation (affiliated or not with another SHO/NGO). Table 2 presents the goodness-of-fit of the five-factor model by each subgroup. The model fit indices were all good: shorter duration subgroup (*n* = 114), CFI = 0.934, TLI = 0.919, RMSEA = 0.075, SRMR = 0.071; longer establishment duration subgroup (*n* = 107), CFI = 0.930, TLI = 0.914, RMSEA = 0.070, SRMR = 0.068; affiliated subgroup (*n* = 115), CFI = 0.929, TLI = 0.914, RMSEA = 0.077, SRMR = 0.062; and non-affiliated subgroup (*n* = 117), CFI = 0.942, TLI = 0.928, RMSEA = 0.067, SRMR = 0.068. Overall, it can be concluded that the five-factor model of the SHODS fitted the data well. 

### 3.3. Reliability and Validity Analysis

Reliability analysis was conducted to evaluate the internal consistency of the SHODS. The Cronbach’s alpha values for each subscale were satisfactory: 0.851 for CS, 0.950 for BS, 0.840 for MRMA, 0.783 for OH, and 0.837 for FS (see Table 3). These results provided evidence for the adequate reliability of the SHODS. The correlations among the five factors are presented in Table 4. As expected, the subscale scores were significantly and positively correlated with each other.

Concurrent validity was tested by correlation analyses. We correlated each factor of the SHODS with each of the five organizational variables. As shown in Table 5, all five factors were significantly and positively correlated to staff supervision, staff understanding, networking, advocating, and educating the public and patients. The concurrent validity and hypotheses were supported by the above findings. 

Based on all the results, we can state that the SHODS is a valid and reliable scale that can be utilized for assessing SHO development in Hong Kong. 

## 4. Discussion

The present study aimed to develop and validate an instrument to measure SHO development. Our results showed that the five-factor structure was consistent with the conceptual framework, as validated by CFA. Furthermore, the Cronbach’s alpha values of the SHOD factors were higher than the recommended cutoff (0.70), demonstrating satisfactory reliability [33]. 

Moreover, despite the smaller sample sizes of the subgroups, subgroup analyses indicate that the CFA model fit results were sufficient across the two pairs of subgroups, namely shorter vs. longer establishment duration and affiliated vs. not affiliated with another SHO/NGO. The results show subgroup consistency and provide further evidence to support the SHODS as a promising assessment tool. The fit indices of shorter and longer establishment duration subgroups were similar. Furthermore, the fit indices were also similar between the subgroups of affiliated vs. not affiliated with another SHO/NGO, with the subgroup of not affiliated having slightly better fit indices. These results indicate that SHODS can be applied to SHOs with different number of years of establishment and SHOs that are either affiliated or not affiliated with other SHOs/NGOs. The results also imply that although the SHODS can be applied to the aforementioned subgroups, SHOs affiliated with other SHOs/NGOs may have other specific indicators of organizational development besides the five factors illustrated in the current study. For instance, cooperation with their affiliated SHOs/NGOs may also be an area of concern for these SHOs.

The correlations among the five factors of the SHODS were consistent with our integrated theoretical framework of the goal attainment model (member recovery and mutual aid, functional sustainability), the internal process model (organizational health), and the system resource model (citizen support, business support) [18,19]. 

Factors under the same theoretical model tended to have higher positive correlations. For instance, the relationship between member recovery and mutual aid and functional sustainability had the strongest positive correlation. Both of these factors fall under the goal attainment model and reflect the SHO’s ability to achieve its goals and objectives [19]. SHOs that are successful in facilitating positive change such as mutual aid and recovery among members are also more effective in serving important functions such as increasing members’ knowledge about the chronic health condition and meeting members’ needs. 

Similarly, the relationship between citizen support and business support had the second-strongest positive correlation. In general, SHOs that receive more support from citizens also receive more support from businesses. These two factors are similar in nature: both involve interactions with external stakeholders and the macro social system. Both factors are explained by the system resource model, in which an SHO is able to gain resources and support from its social environment [18]. 

Citizen support and organizational health had the third-strongest positive correlation, and business support and organizational health had the fourth-strongest positive correlation. This suggests that SHOs with better organizational health and stable resources may be better able to attract citizens and businesses who support and build positive relationships with their organization. It may also suggest that the more citizens and businesses invest in the SHO, the more stable the SHO’s resources. In other words, organizational health and stability explained by the internal process model may not only affect the internal functioning of an SHO but may also indirectly affect the SHO’s external environment as explained by the system resource model. Conversely, it is also possible that interactions with the external environment may influence the internal health of an SHO. 

Concurrent validity was also tested by correlations with five organizational variables. In general, the SHOD factors were significantly and positively correlated with staff supervision, staff understanding, networking, advocating, and educating the public and patients. These findings are in line with the five hypotheses in which the developmental outcomes were predicted to correlate positively with organizational variables. Interestingly, factors under the same theoretical model were found to have similar patterns in their correlations with organizational variables. 

Member recovery and mutual aid specifically refers to members’ sense of hope and resilience, the mutual aid and encouragement among members, as well as members’ stress reduction [23,24]. Member recovery and mutual aid, which is under the goal attainment model, had the strongest correlation relationships with educating the public and patients, followed by staff supervision and advocating. Through raising the public’s awareness and participating in advocacy, members are able to find more strength from the community to be able to reduce their stress, be hopeful about their recovery, and support one another in the SHO [34]. A self-help friendly community can also be built from educating the public and patients and advocating, in which SHO members are encouraged to support one another and create an environment beneficial to members’ recovery [8]. Moreover, SHO staff often makes contact with members and are often involved in the coordination of activities. Thus, adequate staff supervision is important in ensuring that staff is committed to organizational goals and provides good quality support to members [29,35].

Functional sustainability refers to the SHO’s achievements in increasing members’ knowledge about the chronic health condition and their self-care, satisfying members’ needs, and having members affirm their own values [8,21,24,27]. Functional sustainability, which is under the goal attainment model as well, had the strongest correlation with staff supervision, followed by educating the public and patients and advocating. These results had a similar pattern with the results for member recovery and mutual aid. Staff plays an important role in achieving important functions of the SHO that are valued by its members. Adequate staff supervision equips staff with essential skills necessary to achieve these important SHO functions [29]. Furthermore, by being active in educating the public and patients and advocating, members are not only able to actively gain more knowledge about their chronic health condition, but they also become more able to affirm their value by actively promoting change in their community [21,36].

Organizational health refers to SHOs’ financial stability, stability of resources, and adaptability to the changing environment [8,21,24,26]. Organizational health, under the internal process model, had the strongest correlation with networking, followed by staff supervision and staff understanding. An SHO depends on the participation and support of different stakeholders to continue operating and thriving. Relying on members alone to support an SHO may not be sufficient as an SHO develops. Networking with stakeholders in the community increases collaboration opportunities, expands funding sources, and creates synergy by utilizing the expertise of different stakeholders, which improves an SHO’s organizational health as reflected by its stability of resources and adaptability to the changing environment [24,37]. Furthermore, good staff supervision is important to maintain organizational health, as SHO staff provide assistance in administrative tasks such as the management of resources. Ensuring staff understanding of the SHO can prevent issues that may hamper organizational health, such as unclear roles and responsibilities as well as problems with goal alignment [29,38].

Citizen support refers to citizens’ understanding of, monetary support for, and supportive actions for the SHO [20,24,25,28]. Citizen support, under the system resource model, had the strongest correlation relationship with networking, followed by educating the public and patients and advocating. These results indicate that collaborations and interactions with different stakeholders can enhance the public’s awareness of the SHO and increase the SHO’s reputation [20]. To obtain more citizen support, an SHO can consider putting more effort into networking and building collaborative relationships with different community stakeholders, such as the media, businesses, and health care professionals. SHOs should also be active in advocating and educating the public and patients about the SHO and chronic health conditions, which potentially reduces stigmatization and draws public attention to issues related to SHOs [39]. 

Business support refers to businesses’ understanding of the SHO, good relationships with businesses, and businesses’ support for the SHO [20,21,22,24,28]. Business support, under the system resource model as well, had the strongest correlation relationship with advocating, followed by networking and educating the public and patients. These results were similar to those of citizen support. These findings indicate that SHOs involved in external collaborations and activities tend to have greater business support. As advocacy activities make SHOs visible in the community, these activities often attract attention from businesses [40,41]. SHOs that are active in advocacy may have more collaboration opportunities with businesses. Besides collaborative projects, businesses also show their support by providing funding to SHOs on advocacy projects. In turn, businesses increase their exposure to the public, which is a mutually beneficial situation for businesses and SHOs [40].

This study has several theoretical and practical contributions. Theoretically, this study is a first attempt to apply the three organizational development models (goal attainment model, internal process model, and system resource model) to SHOs. The theory-oriented framework proposed in our study offers academics and practitioners an overview of the key components of SHO development and provides guidance on what can be monitored in SHO development. This framework reflects a comprehensive perspective, which means that researchers and practitioners should simultaneously consider the internal resources, external resources, and goal attainment of the SHO. Our theoretical basis is extensively validated, and the five factors included in our framework have the potential to be applied to SHO development internationally. 

Additionally, the SHODS developed and validated in our study can serve as a road map for SHOs in practice. It provides SHO leaders with a tool to identify the specific developmental outcomes of their SHOs, which can help them balance and coordinate the five dimensions. 

The different strengths of the correlation relationships give us insight into how SHOs can improve specific developmental outcomes. Thus, besides using this validated tool to assess the strengths and areas for improvement regarding SHOs’ developmental outcomes, the findings of the correlation analysis will also assist SHOs in planning their activities. For instance, SHOs can put more emphasis on organizational variables that correlate the strongest with developmental outcomes they want to strengthen.

Moreover, organizations or SHOs could also utilize this tool to measure changes in SHOs’ developmental outcomes after participating in training or after implementing new strategies. These organizations can review and modify their training programs or strategies based on the assessment and changes observed in these five developmental outcomes. Furthermore, this tool provides a new assessment tool for funders to gain a better understanding of the developmental outcomes of potential and existing grantees.

Despite these contributions and significance, some limitations need to be noted and addressed by future research. First, data were collected using convenience sampling; thus, the generalizability of our findings is restricted. Future research could adopt other sampling methods to recruit a larger population and a random sample. Moreover, this study focused on core members who play important roles in SHO operation and development. Future research should also collect data from ordinary members.

Another limitation was that data were obtained from SHOs in Hong Kong exclusively which limits the generalizability of the scale. Hong Kong is a densely populated city with an efficient transport system. The characteristics of SHOs in rural areas and interactions among their SHO members may be different from those of the SHOs in Hong Kong. Thus, the SHODS may need to be adapted for the application in rural areas. For instance, the SHODS could incorporate measurements for face-to-face, hybrid, and online services to address the needs of SHOs in rural areas in the future. Moreover, the SHODS should be administered in other regions in future studies to further examine its generalizability.

In addition, some psychometric properties, such as test-retest reliability and discriminant validity, were not examined in this study. These could be tested in future research. Future research could replicate our study and validate whether the five-factor structure can be applied to other regions or other types of SHOs serving different populations. In this study, criteria used to establish the subgroups were SHO establishment duration and organization affiliation. Future studies could explore the application of SHODS to other subgroups such as comparing SHOs with or without their own agency site as well as comparing SHOs that adopt a face-to-face, hybrid, or an online mode of service.

Finally, future studies could compare SHODS results with other assessment methods to enrich the validity testing. For example, the assessment of SHOs’ organizational development by experts in this area could also be obtained. The self-reported SHODS results could be compared with the experts’ assessment to further test for criterion validity of the SHODS [42].

## 5. Conclusions

This study developed and validated a five-factor measurement tool to measure SHO development. Citizen support, business support, member recovery and mutual aid, organizational health, and functional sustainability were the five factors resulting from the CFA. Subgroup analysis demonstrated that the five-factor structure is stable across SHOs with longer or shorter establishment duration as well as SHOs affiliated or not affiliated with other organizations.

The correlations among the five factors of the SHODS and the results of the concurrent validity testing revealed patterns consistent with the integrated theoretical framework of the goal attainment model (member recovery and mutual aid, functional sustainability), the internal process model (organizational health), and the system resource model (citizen support, business support). The results of the concurrent validity testing supported the hypotheses in which organizational variables were significantly and positively related to the five SHO development factors. These results provided insights into how organizational variables are conducive to the five factors of SHO development examined in this study.

In conclusion, results from the CFA, subgroup analyses, correlation analyses, and concurrent validity testing indicated that the SHODS is a novel and promising tool for measuring SHO development.

## Figures and Tables

**Table 1:** Means and standard deviations of the factors in the model.

Factor	*N*	Mean	SD
Citizen support	229	33.93	20.41
Business support	229	36.17	28.88
Member recovery and mutual aid	231	66.47	17.23
Organizational health	230	46.86	19.22
Functional sustainability	230	59.75	16.19

**Table 2:** CFA goodness of fit for total sample and subgroup samples.

Model Fit Indices	CFA of Total Sample Model (*n* = 232)	SHO Establishment Duration in Months (Median = 240)		Affiliation with Another SHO/NGO	
		Below or Equal to Median (*n* = 114)	Above Median (*n* = 107)	Affiliated (*n* = 115)	Not Affiliated (*n* = 117)
CFI	0.959	0.934	0.930	0.929	0.942
TLI	0.950	0.919	0.914	0.914	0.928
RMSEA	0.056	0.075	0.070	0.077	0.067
SRMR	0.052	0.071	0.068	0.062	0.068

**Table 3:** Standardized factor loadings.

Factor	Item	CFA	Cronbach’s Alpha
Citizen support	CS1 Receiving donations from citizens	0.755	0.851
	CS2 Citizens collaborating with the SHO to advocate for patient rights	0.820	
	CS3 Citizens participating in the SHO’s volunteer service	0.827	
	CS4 Citizens having adequate understanding of the SHO	0.693	
Business support	BS1 Having good relationships with businesses and enterprises	0.963	0.950
	BS2 Businesses and enterprises having a good understanding of the SHO	0.946	
	BS3 Receiving funding support from businesses and enterprises	0.877	
Member recovery and mutual aid	MRMA1 Good self-help and mutual support among members	0.712	0.840
	MRMA2 Positive change in members’ sense of hope and resilience in the face of the chronic health condition	0.810	
	MRMA3 Members’ stress has been reduced	0.771	
	MRMA4 Members are appreciated by others	0.716	
Organizational health	OH1 Stable funding	0.652	0.783
	OH2 Stable supplies, such as wheelchairs, computers, and recreational facilities	0.748	
	OH3 Stable human resources	0.778	
	OH4 Adapting to the changing environment	0.579	
Functional sustainability	FS1 Members gain more knowledge about self-care and their chronic health condition	0.801	0.837
	FS2 Members express fulfilment of their needs	0.820	
	FS3 Members recognize their own values	0.762	

**Table 4:** Correlations among the five factors of total sample model.

Factor	Citizen Support	Business Support	Member Recovery and Mutual Aid	Organizational Health	Functional Sustainability
Citizen support	1				
Business support	0.463 ***	1			
Member recovery and mutual aid	0.197 **	0.263 ***	1		
Organizational health	0.362 ***	0.316 ***	0.265 ***	1	
Functional sustainability	0.280 ***	0.199 **	0.555 ***	0.307 ***	1

** *p* < 0.01, *** *p* < 0.001.

**Table 5:** Correlations between the five factors and the organizational variables.

Factor	Organizational Variables				
	Staff Understanding	Staff Supervision	Networking	Advocating	Educating the Public and Patients
Citizen support	0.229 **	0.259 **	0.579 ***	0.542 ***	0.573 ***
Business support	0.166 *	0.262 **	0.463 ***	0.500 ***	0.407 ***
Member recovery and mutual aid	0.276 ***	0.377 ***	0.327 ***	0.349 ***	0.439 ***
Organizational health	0.460 ***	0.467 ***	0.496 ***	0.361 ***	0.393 ***
Functional sustainability	0.283 ***	0.328 ***	0.299 ***	0.307 ***	0.323 ***

* *p* < 0.05, ** *p* < 0.01, *** *p* < 0.001.

## Data Availability

The datasets generated during and/or analyzed during the current study are not publicly available due to datasets containing information that could compromise the privacy of research participants. The data that support the findings of this study are available from the corresponding author (S.S.-y.N.) with the permission of the funder (The Hong Kong Society for Rehabilitation) upon reasonable request.
